# Post-Liver Transplantation Diabetes Mellitus: A Review of Relevance and Approach to Treatment

**DOI:** 10.1007/s13300-018-0374-8

**Published:** 2018-02-06

**Authors:** Maria J. Peláez-Jaramillo, Allison A. Cárdenas-Mojica, Paula V. Gaete, Carlos O. Mendivil

**Affiliations:** 1 Universidad de los Andes School of Medicine, Bogotá, Colombia; 2 Endocrinology Section, Department of Internal Medicine, Fundación Santa Fe de Bogotá, Bogotá, Colombia

**Keywords:** Cyclosporine, Diabetes, Liver transplant, Rejection, Steroids, Tacrolimus

## Abstract

Post-liver transplantation diabetes mellitus (PLTDM) develops in up to 30% of liver transplant recipients and is associated with increased risk of mortality and multiple morbid outcomes. PLTDM is a multicausal disorder, but the main risk factor is the use of immunosuppressive agents of the calcineurin inhibitor (CNI) family (tacrolimus and cyclosporine). Additional factors, such as pre-transplant overweight, nonalcoholic steatohepatitis and hepatitis C virus infection, may further increase risk of developing PLTDM. A diagnosis of PLTDM should be established only after doses of CNI and steroids are stable and the post-operative stress has been overcome. The predominant defect induced by CNI is insulin secretory dysfunction. Plasma glucose control must start immediately after the transplant procedure in order to improve long-term results for both patient and transplant. Among the better known antidiabetics, metformin and DPP-4 inhibitors have a particularly benign profile in the PLTDM context and are the preferred oral agents for long-term management. Insulin therapy is also an effective approach that addresses the prevailing pathophysiological defect of the disorder. There is still insufficient evidence about the impact of newer families of antidiabetics (GLP-1 agonists, SGLT-2 inhibitors) on PLTDM. In this review, we summarize current knowledge on the epidemiology, pathogenesis, course of disease and medical management of PLTDM.

## Introduction

The importance of transplantation as a treatment for many types of organ failure is on the rise. In the USA alone, 33,610 transplant procedures were performed in 2016 and 28,748 were reported to the United Network for Organ Sharing (UNOS) from January to November 2017 [1]. Liver is the second most commonly transplanted organ, representing 23.3% of all transplant procedures [1].

According to the European Liver Transplant Registry, which included more than 93,000 liver transplants (LT) between 1968 and 2009, the most frequent indication for LT is cirrhosis secondary to viral hepatitis or alcohol abuse. After cirrhosis, the most frequent indications are primary liver tumors and cholestatic disease (primary biliary cirrhosis or extra hepatic biliary atresia) [2]. The survival rate of LT patients has improved drastically in recent years, reaching nearly 85% at 1 year and 73% at 5 years in the European registry. The respective rates in the USA are 88% and 70% [3]. The rate of 1-year graft survival currently reaches 80–90% in the USA and Europe and is close to 70% in Brazil and other Latin American countries [4, 5]. Still, nearly 30% of deaths in LT patients are caused by cardiovascular or cerebrovascular diseases [2].

While there has been a great improvement in the survival of LT patients, the world is experiencing a sharp increase in the prevalence of diabetes mellitus (DM). DM is a chronic disease characterized by high levels of plasma glucose secondary to a deficit of insulin activity that results in multiple metabolic disturbances. Approximately 422 million people are known to be affected by DM up to 2014 [6], and this figure is expected to rise worldwide as societies become older, more obese and more sedentary [7]. In 2012, DM caused an estimated 3.7 million deaths, most of them due to cardiovascular diseases (CVD) [8]. DM also leads to significant morbidity associated to chronic complications, such as blindness, kidney failure, lower limb amputation, stroke and non-fatal cardiovascular events [9].

One of the most frequent complications after liver transplantation is the development of post-liver transplantation diabetes mellitus (PLTDM). In addition to all the well-known complications of DM, PLTDM is associated with reduced graft function, increased risk of transplant loss and worsened patient survival [10].

PLTDM can be defined as a degree of hyperglycemia that is consistent with current definitions of DM in a patient who has received a LT. A reliable diagnosis of PLTDM must be made after the patient has been discharged and doses of immunosuppressive agents have been tapered and are stable [11].

The aim of this review is to summarize the essential aspects of the definition, risk factors, pathophysiology and medical management of PLTDM. We therefore performed literature searches in PubMed, Embase, Google Scholar and Scielo using the following terms alone, in combination or as part of Boolean operators: “Liver transplant,” “hepatic transplant,” “transplantation,” “liver graft,” “calcineurin inhibitors,” “tacrolimus,” “cyclosporine,” “steroids,” “rejection,” “diabetes,” “diabetes mellitus,” “hyperglycemia,” “diabetes treatment,” “antidiabetic” and “insulin.” We also consulted the references of prior reviews, consensus reports and meta-analyses on post-transplant DM. We did not have any a priori exclusion criteria based on language or publication date.

### Compliance with Ethics Guidelines

This article is based on previously conducted studies and does not contain any studies with human participants or animals performed by any of the authors.

## Epidemiology of PLTDM

There is wide variation in the reported incidence of PLTDM in the literature. This stems partially from a lack of consensus over the past decades regarding an operational definition and from the use of different criteria by different research groups. International Consensus Guidelines developed in 2014 [11] recommend the use of either the World Health Organization [12] or American Diabetes Association [13] criteria (see section Diagnosis of PLTDM). However, discrepancies in the definition of PLTDM still persist among studies. Additionally, since some cases of PLTDM may be transient, the length of the follow-up and time to diagnosis influence the overall reported incidence [14–17]. When the incidence of PTLDM is normalized by year, studies of shorter duration tend to report higher incidences, while longer follow-up periods yield lower estimates (Table 1). This difference reflects the self-resolving nature of PLTDM in a fraction of patients. The cumulative incidence of PLTDM during the first year after transplantation ranges from 10.8 to 33%, which represents a remarkable burden to any health system, given all the consequences detailed further in this review. The reported average yearly incidence of PLTDM ranges from 3.3 to 30.8% (Table 1).

**Table 1:** Incidence of post-liver transplantation diabetes mellitus in various studies

Reference	Population	Median follow-up (years)	Incidence 1 year post-transplant (%)	Average incidence during follow-up (% per year)	Definition
[19]	902 LDLT; 19,582 DDLT	5.0	12.2	4.6	At least one record of diabetes after transplantation in UNOS. Criteria differed by center.
[14]	161 LDLT	4.2	10.8	3.3	ADA/WHO 2003 [18]
[20]	15,463	1.9	21.7	14.1	At least one record of diabetes after transplant in UNOS. Criteria differed by center.
[15]	430	1.0	19.0	19.0	Two RPG measurements ≥ 200 mg/dL at least 30 days apart or use of antidiabetic medications for ≥ 30 consecutive days or HbA1c ≥ 6.5%
[35]	763	2.6	33.0	13.5	Two FPG measurements ≥ 126 mg/dL at least 30 days after transplantation or use of antidiabetic agents
[21]	115 LDLT	2.9	26.1	11.1	ADA/WHO 2003, excluding cases diagnosed in first 3 months after transplant [18]
[30]	158	4.7	NR	8.3	De novo and persistent hyperglycemia requiring long-term treatment with antidiabetic medications.
[22]	169	1.0	30.8	30.8	ADA/WHO 2003 [18]
[31]	225	1.0	17.0	17.0	ADA 2010. Patients still on corticosteroids 4 months after transplant were excluded.
[27]	364	3.6	16.9	5.3	ADA/WHO 2003 or HbA1c ≥ 6.5% [18]
[16]	555	5.0	NR	1.9 (3.6 for transient DM)	Use of antidiabetic medication or DM diagnosis in medical record
[24]	18,741	3.1	13.0	9.5	ADA/WHO 2003 [18]
[25]	10,204	2.6	22.9	9.3	ADA/WHO 2003 [18]

*ADA* American Diabetes Association, *DDLT* deceased-donor liver transplant,* DM* diabetes mellitus, *FPG* fasting plasma glucose,* HbA1c* glycated hemoglobin A1c, *LDLT* living-donor liver transplant, *NR* not reported, *RPG* random plasma glucose, *UNOS* United Network for Organ Sharing,* WHO* World Health Organization

### Risk Factors for PLTDM

**Fig. 1:**
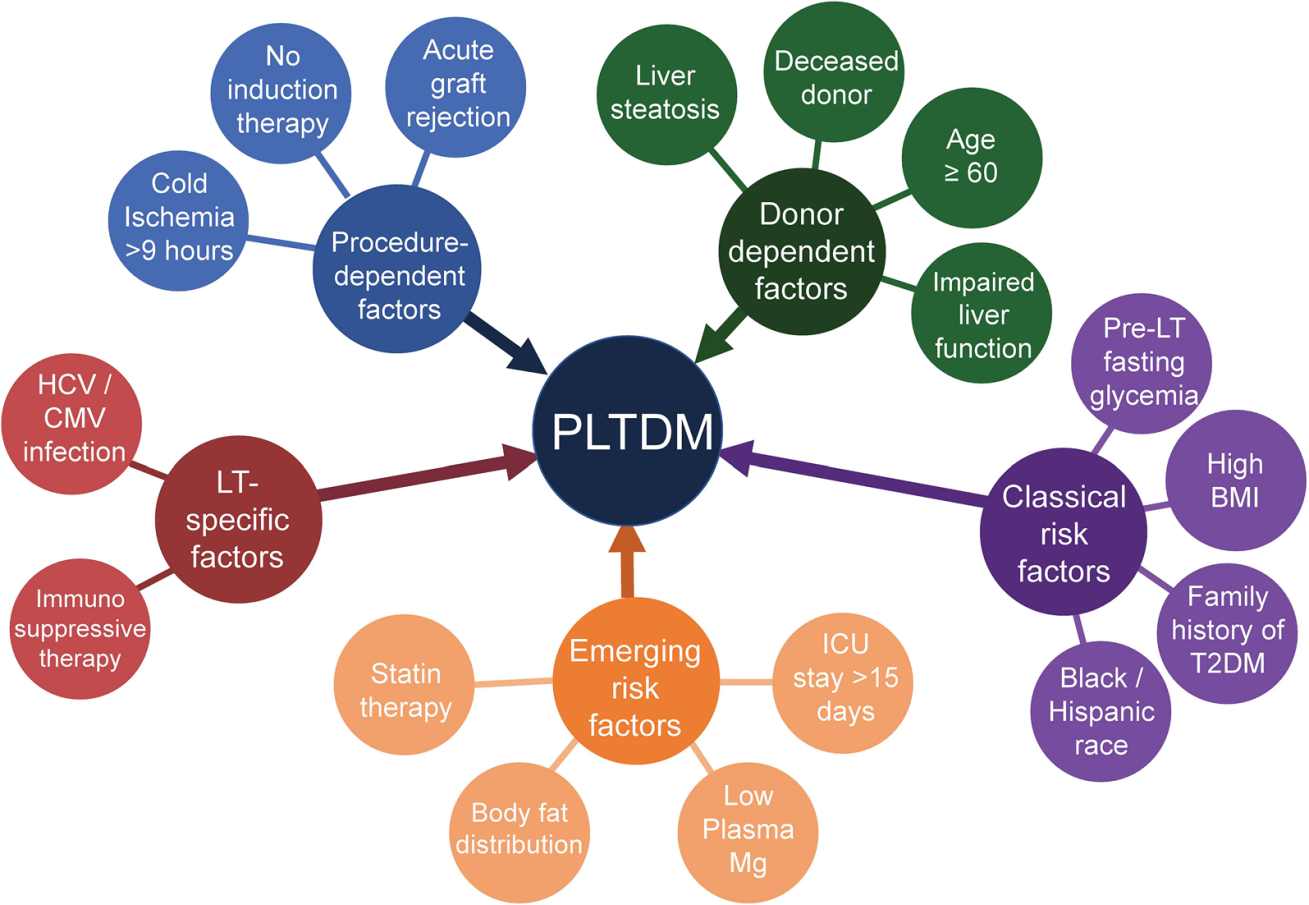
Risk factors for the development of post-liver transplant diabetes mellitus (PLTDM). *HCV* Hepatitis C virus, *CMV* cytomegalovirus, *LT* liver transplant, *ICU* intensive care unit,* BMI* body mass index,* T2DM* type 2 DM

Risk factors for PLTDM can be classified into two groups: those associated with the development of DM in the general population and those specifically associated with increased DM risk among LT recipients (Fig. 1).

In the first group, “classical” risk factors for PLTDM with robust evidence support include older age [16–25], male sex [17, 19, 24, 25], high body-mass index (BMI) [19, 20, 24, 25], pre-transplant impaired fasting glucose [26–28], family history of DM [26] and African American or Hispanic ethnicity [20, 29].

Conditions that predispose particularly to development of DM after a LV include hepatitis C virus (HCV) [15–20, 24, 26, 28, 30–33] or cytomegalovirus [22, 25] infection and immunosuppressive therapy with high-dose corticosteroids [14, 15, 20, 24, 25, 34] or calcineurin inhibitors (CNIs; tacrolimus or cyclosporine) [16, 20, 21, 23, 24, 26, 35, 36]. Nonalcoholic steatohepatitis (NASH) is a strong risk factor for the development of type 2 diabetes (T2DM) in the general population [37], and there is no reason to believe that this association would be any different in patients who have received a LT.

Some less well-established determinants of PLTDM in the recipient are statin therapy [27], central body fat distribution prior to transplantation [32], low magnesium levels before and 1 month after surgery [22], hyperglycemia in the first post-transplant month [25, 36] and stay in the intensive care unit (ICU) > 15 days [25].

Donor characteristics also play a key role in predisposing or protecting from PLTDM. Factors associated with increased risk are age > 60 years [19, 20], male gender [25], computed tomography scan- or biopsy-diagnosed liver steatosis [14, 35] and deceased liver donor [15, 25]. In a study of Japanese recipients of living donor liver transplants, cholinesterase plasma levels of < 185 IU/L (as a measure of liver function in the donor) were an independent risk factor [21].

Among the variables related to the transplant procedure itself, a cold ischemia time of > 9 h [25] is detrimental. The use of induction therapy with agents other than corticosteroids as part of the immunosuppressive regime has been found to be a protective factor against PLTDM in several studies, two of which used basiliximab, a monoclonal antibody directed to the interleukin-2 receptor [20, 24]. Acute graft rejection also predisposes to PLTDM [19, 22, 24, 38], but a causal link is hard to establish given that acute rejections are usually treated with high doses of corticosteroids, which induce hyperglycemia [30].

## Pathogenesis of PLTDM

### CNIs and PLTDM

Calcineurin inhibitors are a family of highly effective immunosuppressive drugs that have revolutionized transplantation medicine over the last 40 years. Both cyclosporine and tacrolimus were developed by multidisciplinary research teams working at pharmaceutical companies and searching for immunosuppressants with a mild profile of cytotoxic adverse effects [39]. Cyclosporine is a hydrophobic cyclic undecapeptide with N-methylated amino acids that render it resistant to digestion by gastrointestinal system proteases [40, 41]. Tacrolimus is a macrolide antibiotic with a slightly better water solubility than cyclosporine. After intestinal absorption and entry into cells, both of these CNIs bind a cytoplasmic protein belonging to the immunophilin family: cyclophilins in the case of cyclosporine and FK-binding protein (FKBP) in the case of tacrolimus [41].

**Fig. 2:**
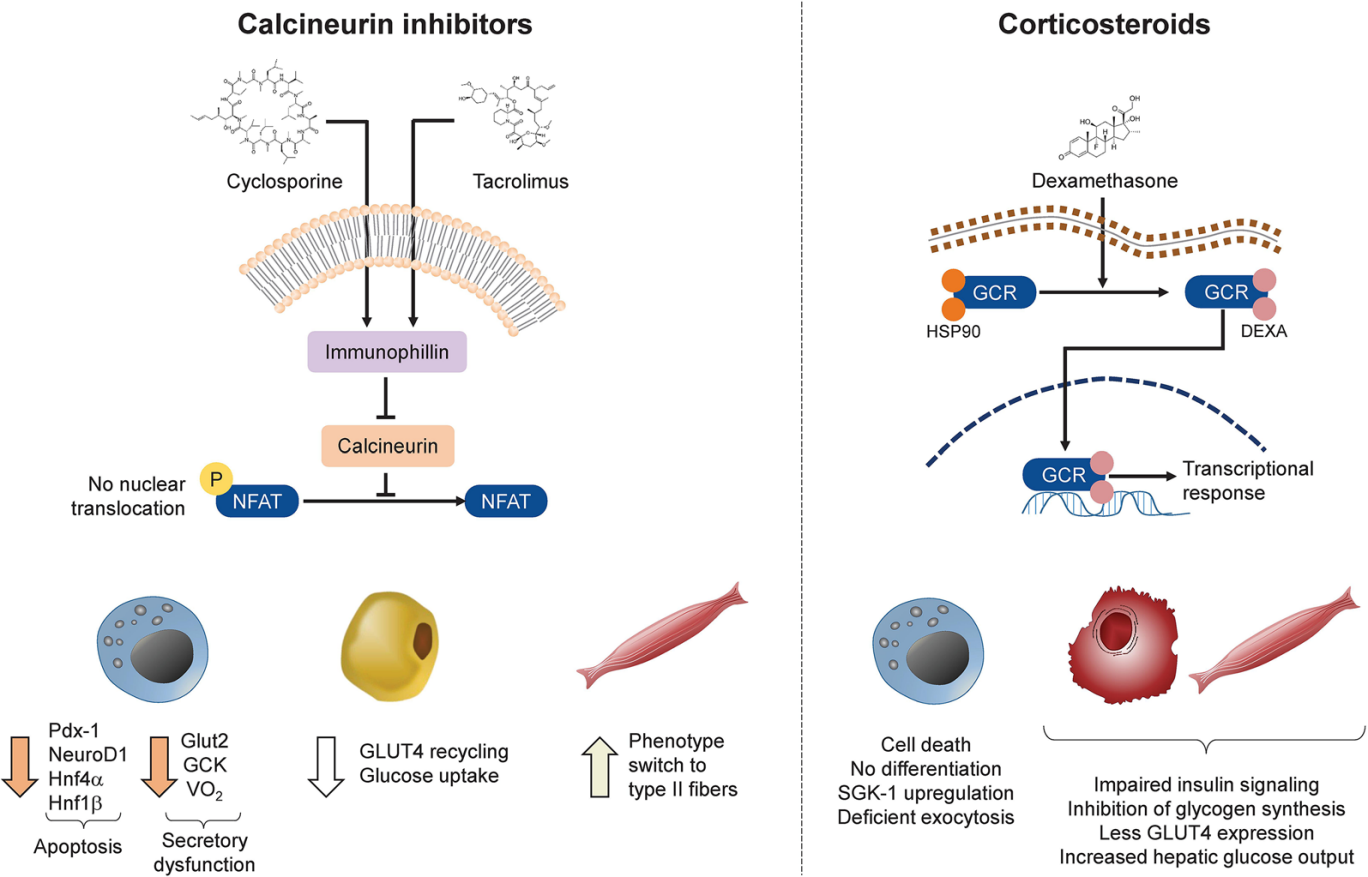
Pathogenesis of PLTDM/diabetogenic mechanisms of immunosuppressive drugs frequently employed in liver transplant (LT) patients. Calcineurin inhibitors (CNIs) bind to one of the immunophilins (cyclophylins in the case of cyclosporine and FK506-binding proteins [FKBPs] in the case of tacrolimus), which in turn inhibit calcineurin and prevent the dephosphorylation and nuclear translocation of the transcription factor nuclear factor of activated T-cells (*NFAT*). As a consequence, expression of key transcription factors involved in beta-cell survival is reduced, including pancreatic and duodenal homeobox 1 (*Pdx-1*), neurogenic differentiation 1 (*neuroD1*), hepatocyte nuclear factor 4 alpha (*Hnf4-α*) and hepatocyte nuclear factor 1 beta (*Hnf1-β*). Lack of NFAT nuclear translocation also causes a reduction in the expression of the glucose transporter 2 (*GLUT2*) and glucokinase (*GCK*) genes and in mitochondrial oxygen consumption (*VO*_*2*_). In adipose tissue, less NFAT activity reduces recycling of glucose transporter 4 (*GLUT4*) and subsequently glucose uptake. In muscle tissue, blockage of NFAT induces a phenotype switch from type I (slow-twitch) to type II (fast-twitch) fibers. Corticosteroids (dexamethasone is used here as an example) bind to their receptor in the cytoplasm, inducing the release of the chaperone heat shock protein 90 (*HSP90*) and nuclear translocation of the glucocorticoid–receptor complex (*GRC*). This complex acts as a master regulator of the expression of multiple genes. The ultimate effects of this transcriptional response in beta-cells entail deficient exocytosis of insulin granules and eventually apoptosis. In liver and muscle cells, the transcriptional response to glucocorticoids impairs insulin action, resulting in less glucose uptake, reduced glycogen synthesis and increased gluconeogenesis

The cyclosporine–cyclophilin or tacrolimus–FKBP complex inhibits calcineurin, a calcium-dependent phosphatase involved in T-cell activation and regulation via dephosphorylation of nuclear factor of activated T-lymphocytes (NFAT) [42, 43]. However, calcineurin and NFAT are relevant not only in immune cells but also in many other tissues, including kidney, heart, spleen, liver, testes, brain and pancreas [44, 45]. In pancreatic beta-cells, calcineurin promotes the transcription of survival factors and stimulates the growth and expansion of the beta-cell mass [46]. Calcineurin is also involved in metabolic signaling pathways in adipose [47] and skeletal muscle tissue [48]. Widespread inhibition of calcineurin by CNIs during immunosuppressive therapy [45] may therefore interfere with its action in all these tissues and lead to potential metabolic side effects (Fig. 2).

The mechanistic factors by which CNIs contribute to the development of PLTDM are diverse and include deregulation of insulin secretion, apoptosis of insulin-producing beta cells and induction of peripheral insulin resistance. Studies in cultured beta-cells have reported a reduction of basal and glucose-stimulated insulin secretion after exposure to cyclosporine, an effect less consistently found with tacrolimus [49, 50]. In addition to the inhibition of calcineurin [51], other targets have been implicated in CNI-induced insulin secretory dysfunction. Of note, tacrolimus treatment has been observed to reduce the number and oxygen consumption of beta-cell mitochondria in vitro, limiting the availability of ATP and other metabolites essential for secretory pathways [52]. Similarly, studies in isolated murine islets revealed cyclosporine-mediated inhibition of the permeability transition pore, a mitochondrial protein important for the regulation of cytoplasmic calcium oscillations and therefore vesicle-mediated exocytosis [53]. Tacrolimus also affects the normal closure of ATP-sensitive potassium channels [54] and reduces glucokinase (but not hexokinase) activity [55].

Experiments with reporter genes in hamster beta cells found that CNIs suppress the transcription of genes whose promoters contain cAMP-responsive elements [56], many of which are essential to the survival, replication and function of beta-cells [57, 58]. In fact, the administration of tacrolimus to Sprague–Dawley rats negatively impacts their beta cell mass [59]. The impact of CNIs on peripheral insulin action does not seem to be as crucial as their impact on beta-cells, but calcineurin inhibition does have an effect on insulin sensitivity. When primary human adipocytes were incubated with high concentrations of a CNI (100 nM), the amount and phosphorylation of key intermediaries of the insulin signaling pathway (IRS1/2, p85-PI3 K, PKB, AS160, and mTORC1) did not change [60], but the membrane content of glucose transporter 4 (GLUT4) transporters and the uptake of C14-labeled glucose did diminish. This effect was apparently due to a slower vesicle-to-plasma membrane recycling of GLUT4. Finally, calcineurin inhibition seems to promote transformation of type I skeletal muscle fibers to less insulin-sensitive type II fibers [61] (Fig. 2).

Clinical data suggest that the impact of specific CNIs on glucose metabolism may not be the same. A meta-analysis of 16 studies involving 3813 patients found a greater degree of glucose impairment with tacrolimus than with cyclosporine [62]. A more recent meta-analysis estimated a PTDLM risk ratio of 0.60 (95% confidence interval [CI] 0.47–0.77) for cyclosporine relative to tacrolimus [63].

### Corticosteroids and PLTDM

Corticosteroids continue to be part of the standard treatment for immunosuppression during the early post-transplant period, and they are known to promote hyperglycemia through a host of different mechanisms. The corticosteroid dexamethasone exhibits cytotoxic [64] and anti-proliferative [65] effects on beta-cell lines in vitro. Also, in vitro exposure to corticosteroids impairs insulin secretion, an effect mediated via the upregulation of serum-and-glucocorticoid-inducible kinase-1 and deterioration of membrane depolarization necessary for glucose-mediated vesicle exocytosis [66]. Corticosteroids interfere with the insulin signaling pathway, thereby reducing insulin-mediated glycogen synthesis [67], GLUT4 translocation and glucose uptake in muscle [68]. Lastly, corticosteroids increase hepatic glucose output, further contributing to fasting hyperglycemia [69] (Fig. 2).

### Mammalian Target of Rapamycin Inhibitors and PLTDM

Mammalian target of rapamycin (mTOR) inhibitors exert their immunosuppressive action by forming a complex with FKBP (the target of tacrolimus) and inactivating the protein mTOR. Inhibition of the mTOR signaling pathway leads to a decrease of cytokine-mediated T-cell activation and proliferation [70]. Only everolimus is approved by both Federal Drug Administration (FDA) and European Medicines Agency (EMA) for use in LT recipients. However, sirolimus is still used in certain LT patients [71]. The metabolic effects reported for these agents are mainly hypertriglyceridemia and hypercholesterolemia [72, 73]. The impact of sirolimus on glucose regulation seems to be less pronounced than that of CNIs. A study in 23 LT patients evaluated the impact of conversion to sirolimus after 4 weeks of CNI therapy. Three patients had developed PLTDM after the use of CNI and had insulin requirements between 80 and 130 IU per day. After conversion to sirolimus, daily insulin requirements dropped to 24–32 IU while blood glucose levels remained stable [74]. A review of data from 227 patients with hepatocarcinoma as an indication for liver transplantation showed a significantly higher incidence of PLTDM in patients treated with tacrolimus + mycophenolate (12.3%) versus those treated with sirolimus (0%; *p* < 0.001) [75].

### Common Origins for Serious Liver Disease and PLTDM

Another hypothesis to explain the high coincidence of DM and LT is that liver diseases for which a transplant is required and DM have a common origin. HCV is both the most common cause of LT in the USA (affecting almost 50% of the recipients) and a risk factor for DM: a meta-analysis showed that HCV infection increased DM by a factor of 1.7 in both retrospective and prospective studies [76]. HCV infection is positively associated with the homeostasis model assessment-insulin resistance (HOMA-IR) index in humans [77], probably due to impairment of insulin signaling in hepatocytes [78, 79]. Mice transgenic for the HCV core gene exhibit overexpression of tumor necrosis factor-alpha (TNF-α), and TNF-α-dependent insulin resistance [80]. Similarly, alcoholic liver disease is the second-most common indication for LT [81] and a DM risk factor: a recent dose–response meta-analysis with over 1.9 million individuals concluded that alcohol intakes of > 120 g/day significantly increase the risk of DM [82].

NASH is another common cause of LT, and the percentage of LT secondary to this condition is estimated to have increased 35-fold between 2000 and 2005 in the USA [81]. NASH is strongly associated with metabolic disturbances known to be risk factors for DM, among which are overweight and abdominal obesity [83]. However, NASH in and of itself is a risk factor for DM and CVD [84], and many patients develop liver steatosis de novo after LT [85]. Thus, there is a bidirectional relationship between NASH and DM, also in the post-LT patient.

## Course of PLTDM

### Persistence of PLTDM

Not all cases of PLTDM persist over time; some resolve spontaneously. There is substantial disparity in the reported persistence of PLTDM across studies, probably due to the heterogeneity of diagnostic criteria and differences in follow-up length [10, 14, 86, 87]. In a study of 17,184 adult LT recipients followed for 5 years, 29.2% developed at least one episode compatible with PLTDM, but persistence for > 1 year was only 4.9% of the initially transplanted patients (7.6% for recipients with NASH) [88]. Characteristics associated with persistent PLTDM include African American race, HCV infection, NASH in the recipient, higher MELD score and acute cellular rejection [87, 89]. The authors of a long-term study of renal transplantation proposed a classification according to the temporal pattern of presentation, as follows: early-onset persistent DM (present in the first year of transplantation and during the next 7 years), late-onset DM (occurring after the first year) or transient DM (diagnosed within the first year but eventually recovering to normal glycemia) [90]. The implications of this temporal classification on the management, prognosis and survival of LT recipients have not yet been established [91].

### Influence of PLTDM on Clinical Outcomes after LT

#### Overall Survival

In an analysis of 798 LT patients from the U.S. National Institute of Diabetes and Digestive and Kidney Diseases registry, pre-LT diabetes (hazard ratio [HR] 1.94, CI 1.40–2.68) and post-LT diabetes (HR 1.87, CI 1.41–2.48) were predictors of death after 1 year [92]. Furthermore, in a 5-year follow-up study of deceased-donor liver transplant recipients, total mortality in patients with persistent PLTDM was 36.5% compared to 13.9% in those with transient PLTDM [93]. A Chinese study of 438 LT patients free of DM before receiving the transplant found a mean survival of 4.2 years in recipients who developed PLTDM, compared to 6.1 years in those without PLTDM (*p* < 0.001) [10]. Likewise, a study of 35,870 LT patients from the U.S. Scientific Registry of Transplant Recipients found a significant independent association between both pre-LT DM and PLTDM and total mortality in the period between 1994 and 2013 (*p* < 0.001 and *p* = 0.004, respectively) [94]. Nonetheless, not all studies have found an increased mortality rate among PLTDM patients. Data from a national registry of LT in Taiwan revealed comparable 11-year survival rates in patients with PLTDM and those without DM [95]. Despite the heterogeneous nature of the evidence, most studies have documented a significant increase in total mortality associated with PLTDM.

### Cardiovascular Disease

Cardiovascular complications are a major cause of non-liver-related mortality after LT, representing 13–28% of all deaths [96–98]. PLTDM has been identified as an independent predictor of post-transplantation CVD events. The CVD rates of patients after a LT were evaluated in a retrospective analysis of the Organ Procurement and Transplantation Network/UNOS database. A comparison of patients with persistent PLTDM, transient PLTDM, pre-LT DM or LT without DM, respectively, revealed that those with persistent PLTDM exhibited the highest risk (HR 1.95 vs. non-DM; *p* < 0.01) [87]. DM at 1 year after LT is much more frequent among recipients who develop CVD events than among those who do not (64 vs. 38%; *p* < 0.001) [99]. Therefore, the occurrence of PLTDM in LT recipients puts this group in a special condition of cardiovascular vulnerability.

### Acute Rejection and Graft Failure

In a small single-center matched case–control study, PLTDM was associated with higher rates of acute rejection, but not with long-term graft failure [100]. Yet in a larger, multicenter prospective study, patients with PLTDM showed increased risk for graft failure [93]. PLTDM is also linked to a higher number of rejection episodes [101]. Data from a large U.S. cohort showed that pre-transplant DM, PLTDM and acute rejection were significantly associated with increased risks for graft failure. However, after multivariate Cox regression adjustment, the association between PLTDM and adverse outcomes did not persist [102]. An important consideration is that the impact of PLTDM on survival of the transplanted organ may be underestimated: patients with PLTDM may develop rejection, graft failure and subsequent death. In these patients, however, the only recorded outcome is mortality. Due to these complexities, it is not possible based on current evidence to ascertain whether or not PLTDM has an independent effect on the risk of graft rejection or failure.

### Infections and Other Complications

In addition to the above-mentioned adverse outcomes, PLTDM patients also have a significantly higher incidence of renal insufficiency and post-operative bacterial infection [10]. In a study of adults who required LT due to HCV infection in the USA, patients with PLTDM developed more HCV recurrences (59%) and stage 2 fibrosis than both patients with pre-LT DM and normal patients [103]. A similar study in the UK found PLTDM to be a strong independent risk factor for the development of a fibrosis score of ≥ 4 over a 6-year follow-up (HR 3.28; *p* = 0.004) [104]. In summary, infections and infection-related complications [26] seem to be higher in patients with PLTDM.

## Diagnosis of PLTDM

In general, diagnostic criteria for PLTDM are the same as those for diabetes in the general population. Because postprandial hyperglycemia is much more prevalent than fasting hyperglycemia among LT patients [105], the ideal screening test for PLTDM is the oral glucose tolerance test (OGTT) [106]. Due to blood losses associated with the transplant, to preexisting anemia due to impaired renal function, and especially to a lack of evidence concerning its use in the early post-transplant period, HbA1c is not recommended as a first-line diagnostic test for PLTDM [100]. A fasting plasma glucose level of < 100 mg/dL (5.5 mmol/L) is considered to be normal, 100–125 mg/dL (5.5–6.9 mmol/L) is considered to be impaired fasting glucose (IFG) and ≥ 126 mg/dL (7 mmol/L) constitutes diabetes. A 2-h post-OGTT plasma glucose level of < 140 mg/dL (7.7 mmol/L) is considered to be normal, 140–199 mg/dL (7.7–11.1 mmol/L) is considered to be impaired glucose tolerance (IGT) and ≥ 200 mg/dL (11.2 mmol/L) constitutes diabetes. The importance of diagnosing the pre-diabetic states (IFG and IGT) in the post-LT context lies in their relevance as predictors of future PLTDM risk.

## Prevention of PLTDM

As in the general population, lifestyle modifications are the cornerstone of PLTDM prevention in LT recipients. The positive association between post-transplant BMI increment and risk of subsequent DM [107] justifies advising weight loss to LT recipients, although this should not be encouraged immediately after surgery to avoid a negative effect on wound healing [106]. Exercise or an increase in daily life physical activity should also be recommended [106]. These measures should be stressed in patients in whom risk factors for PLTDM are identified [11].

An international consensus recommended screening for post-transplant DM using postprandial glycemia and HbA1c, especially in patients at high risk [11]. The consensus also established that there is insufficient evidence to recommend oral antidiabetic agents for prevention in patients with impaired glucose tolerance [11].

## Management of PLTDM

### Importance of Early Glycemic Control

A retrospective review of 184 LT recipients showed that strict intraoperative glycemic control (< 150 mg/dL; actual mean glucose 135 mg/dL) results in a remarkably lower 1-year mortality compared with less strict control (≥ 150 mg/dL; actual mean glucose 184 mg/dL) (8.8 vs 21.9%; *p* = 0.05) [108]. In the same report, patients in the less strict group experienced a cumulative infection rate of 48%, compared to 30% to those in the tighter control group (*p* = 0.02) [108]. In a separate study in a tertiary care transplantation center, the attainment of a perioperative blood glucose level that was lower by 31 mg/dL was accompanied by a significant reduction in infection rates in LT patients (adjusted odds ratio [OR] 0.22, 95% CI 0.06–0.86) [109]. In a large case series, severe intraoperative hyperglycemia (≥ 200 mg/dL) during liver transplantation was an independent risk factor for postoperative surgical site infection (OR 2.25; 95% CI 1.26–4.03; *p* = 0.006) [110].

Poor glycemic control has also been related with longer stays the ICU on the first admission (5.6 vs. 3.2 days; *p* = 0.039) [109] and prolonged ventilation (OR 4.3, 95% CI 1.28–14.4) [104]. Lastly, a retrospective review of 144 liver and liver–kidney transplants found lower rejection rates in patients with in-hospital average glucose levels of < 200 mg/dL (35.1 vs 76.7%, *p* < 0.001) [111].

### Immediate Post-Transplant Period

Several factors increase whole-body insulin requirements during the early post-transplant period, among them immunosuppression with steroids, acute pain and surgical stress [112]. Consequently, intravenous or intensive (three or more injections a day) insulin therapy is the standard of care during the immediate post-transplant phase. The safety of bedside glucose-based, sliding-scale intravenous insulin schemes in hospitalized LT recipients has been documented [113]. Nonetheless, it should be stressed that such schemes demand careful and frequent glucose monitoring by nursing personnel. Once patients have returned to a regular eating pattern, they can be transitioned into a subcutaneous basal/bolus regimen. For patients not receiving total parenteral nutrition, an initial total daily dose of between 0.2 and 0.4 U/Kg is reasonable, of which 50% should be administered as basal insulin and 50% as prandial insulin. Prandial (fast or ultra-fast acting insulin) is used at each meal, usually in a fixed ratio of dietary carbohydrate to insulin. A good initial ballpark is 1–2 U per each 15 g carbohydrate. Supplemental doses of fast-acting insulin should be administered when blood glucose measurements are outside treatment goals [114]. A practical initial approach is to measure blood glucose every 4–6 h and to administer 1–2 U of fast-acting insulin for every 40–50 mg/dL that the blood glucose is above 140–150 mg/dL.

### Treatment Goals

While the transplanted patient is still hospitalized, capillary glucose goals are the same as those for other hospitalized patients: 140–180 mg/dL in the ICU, and < 140 outside the ICU for pre-meal and < 180 mg/dL for 2-h post-meal or random measurements [114]. For the PLTDM outpatient, capillary glucose goals are 70–110 mg/dL for pre-meal measurements and 70–140 mg/dL for post-meal measurements. Ambulatory HbA1c goals can be defined according to diabetes duration, presence of comorbidities and risk of side effects associated with antidiabetic therapy (i.e. hypoglycemia). Thus, an HbA1c of < 7% is a suitable goal for most PLTDM patients. For patients with a short disease duration, younger age and few comorbidities aside from their liver problems, an HbA1c of < 6.5% is both feasible and desirable. For patients with advanced age, multiple comorbidities, high risk of hypoglycemia and/or limited life expectancy, an HbA1c of < 8.0% is a more realistic and potentially less iatrogenic objective. Despite a paucity of long-term studies evaluating the impact of glycemic control in patients with PLTDM, a study in LT recipients with HCV infection found that patients with tight glycemic control (< 138 mg/dL) had a lower rate of Stage 2 fibrosis development relative to patients with a glycemic average above 138 mg/dL (78 vs. 60%; *p* = 0.027) [103].

Other relevant treatment goals include CVD risk factors other than glycemia, as CVD continues to be a major cause of mortality among LT patients in general and PLTDM patients in particular [115]. Smoking should be strongly discouraged, and support for smoking cessation should be provided when needed. The level of low-density lipoprotein (LDL) cholesterol should be kept at < 100 mg/dL in PLTDM patients without prior clinical CVD and at < 70 mg/dL in those with clinical CVD. For patients with progressive CVD despite receiving cholesterol-lowering therapy, an LDL cholesterol level of < 55 mg/dL is recommended by some guidelines [116]. The goal for plasma triglycerides is < 150 mg/dL. However, the management of plasma triglycerides may be particularly difficult in patients who receive the mTOR inhibitors sirolimus or everolimus, as these agents are clearly associated with marked hypertriglyceridemia [117]. Since most PLTDM patients will be receiving a statin for LDL cholesterol management, it must be borne in mind that fenofibrate is the fibrate of choice for combined therapy with statins. Blood pressure goals can be set according to the most recent American Heart Association guidelines [118] to < 130/80 mmHg. Patients with PLTDM, like all other patients with diabetes, must undergo periodic evaluations of eye and foot care, as they are not only at increased risk of the usual complications of DM, but also are particularly prone to cataracts (due to corticosteroid use) and soft tissue infections (due to prolonged immunosuppression).

### Nonpharmacological Interventions

The 2014 international consensus guidelines on post-transplantation DM recommend a stepwise approach for the management of late post-transplantation DM that consists of lifestyle modification followed by oral anti-diabetic therapy and then insulin therapy [11]. Different factors influence the decision of which oral anti-diabetic drug should be the first choice, and every patient should be evaluated individually.

Maintenance of caloric balance and control of body weight are essential components of diabetes management. In patients with kidney transplants, weight gain during the months following transplantation is proportional to the risk of new-onset diabetes, independent of the pre-transplant BMI [119]. Also in kidney transplant recipients, a 6-month intensive lifestyle modification program that included referral to a dietitian, a structured exercise program and weight loss advice induced regression to normoglycemia in up to 44% of IGT patients [120]. Randomized trials are needed in which the overall impact of structured diet and exercise can be specifically assessed in the PLTDM population.

### Pharmacological Interventions

#### Metformin

Metformin controls hyperglycemia by reducing hepatic glucose production and improving peripheral insulin sensitivity without weight gain or hypoglycemia [121]. Even though metformin is the first-line treatment for T2DM [122], its use in PLTDM is still not widely advised due to a relative scarcity of evidence [11]. Part of the reluctance to recommend metformin is based on a perceived increase in the risk of lactic acidosis associated with renal and hepatic disease. However, the translation of this biological plausibility into clinical outcomes has been challenged by several studies. In a retrospective study of T2DM patients treated with metformin, 36 patients developed liver disease and continued to receive the medication, yet not a single one of them developed lactic acidosis [123]. It has actually been shown that continuation of metformin after a diagnosis of cirrhosis is a predictor of better survival compared to discontinuation on the grounds of hepatic disease [124]. Despite the lack of clinical trials in LT patients, in one study there were no cases of lactic acidosis and no decrease in glomerular filtration rate (GFR) with metformin use in kidney transplant recipients [125].

#### Sulfonylureas

There are very few studies evaluating sulfonylureas (SUs) in patients with post-transplant diabetes, but their use has continued empirically for many years. SUs directly stimulate insulin release by closing the ATP-dependent K^+^ channel in pancreatic beta-cells, irrespective of concurrent plasma glucose. This mechanism may result in hypoglycemia, weight gain [126], beta-cell death [127] and progressive loss of efficacy [128]. Another significant pitfall to the use of SUs in PLTDM is the potential for drug–drug interactions due to shared hepatic metabolism pathways with other medications commonly used in this type of patient [129]. Nonetheless, one study showed that glipizide did not alter cyclosporine concentration in renal transplant recipients [130]. Altogether, there is insufficient evidence to recommend SUs as a rational treatment choice in PLTDM.

#### Meglitinides

Repaglinide and nateglinide induce insulin secretion in a glucose-dependent manner. Their rapid onset and short duration of action reduces the risk of hypoglycemia compared to the SUs [131]. Meglitinides undergo extensive hepatic metabolism, which may be an indication that caution is warranted in patients with PLTDM [129]. Nevertheless, their overall efficacy and safety have been documented in renal transplant patients [132, 133]. In five patients with chronic viral hepatitis, repaglinide did not increase transaminase levels [132].

#### Thiazolidinediones

Rosiglitazone and pioglitazone are agonists of peroxisome proliferator-activated receptor gamma (PPAR-γ), a transcription factor mostly expressed in adipose tissue. Thiazolidinediones (TZDs) improve insulin sensitivity and lower plasma triglycerides [134]. Two small studies in solid organ recipients with pre- or post-transplant diabetes evaluated the efficacy and safety of TZD [135, 136]. Both studies added a TZD to insulin, glyburide or repaglinide treatment and found a sizable reduction in HbA1c level (1.2–1.3%) and insulin dose (10–40 U/day) over 8–12 months, with no changes in plasma creatinine or CNI levels. A larger study was performed with rosiglitazone in 32 liver and eight kidney recipients with post-transplant diabetes. Body weight and plasma creatinine and transaminases were stable over the 26-week follow-up. Of the 33 patients initially on insulin, 30 no longer required it at the end of the study period. The percentage goal (HbA1c < 7%) achievement was 30% with rosiglitazone monotherapy and 62.5% with combined rosiglitazone + SU therapy [137]. Despite these encouraging results, the adverse cardiovascular profile of rosiglitazone has prompted its withdrawal from several markets in the world [138]. Meanwhile, pioglitazone has been reported to reduce all-cause mortality, non-fatal myocardial infarction and stroke in patients with DM [139], and it is available in many countries.

#### DPP-4 Inhibitors

Dipeptidyl peptidase-4 (DPP-4) inhibitors prolong the half-life of endogenous incretins (glucagon-like peptide-1 [GLP-1] and glucose-dependent insulinotropic peptide [GIP]), resulting in an antihyperglycemic effect [140]. Interest in DPP-4 inhibitors for the management of PTLDM has surged in recent years, as incretins counteract the diabetogenic actions of immunosuppressant drugs [141]. Notably, increased levels of DPP-4 have been reported in LT patients with HCV but not other etiologies [142].

Evidence supporting the use of DPP-4 inhibitors in post-transplant diabetes comes mainly from kidney recipients. Linagliptin [143], vildagliptin [144] and sitagliptin [145] lower HbA1c levels to 0.3–0.6% without significant alterations in the GFR, immunosuppressant levels or liver function tests. A descriptive study of 65 kidney recipients on different DPP-4 inhibitors found linagliptin to have the greatest effect on HbA1c and the smallest effect on cyclosporine levels [146]. DPP-4 inhibitors have also been used in combination with other agents. In one study 45 kidney recipients with diabetes on metformin were randomized to sitagliptin or insulin glargine. After 12-weeks, safety and efficacy were comparable between groups, but sitagliptin was associated with weight reduction [147].

#### GLP-1 Analogues

Glucagon-like peptide-1, a gastrointestinal hormone of the incretin family, possesses not only antidiabetic but potentially antihypertensive, anti-inflammatory, anti-apoptotic and immunomodulatory actions as well [148]. GLP-1 analogues bind the GLP-1 receptor but are resistant to DPP-4 degradation. These agents lack hepatic metabolism and hence have limited drug–drug interactions, but they do slow gastric emptying, potentially impairing immunosuppressant absorption [129]. No clinical trials of GLP-1 in patients with PLTDM have been reported. One case series of five kidney transplant recipients with post-transplant diabetes reported the safety of liraglutide administered for 21 days, without changes in tacrolimus levels [149]. In the same study, glucose levels at 60 and 120 min after an OGTT were lower with liraglutide at day 21 compared to baseline.

#### SGLT2 Inhibitors

The sodium-glucose cotransporter type 2 (SGLT2) mediates the reabsorption of glucose in the proximal tubule of the nephron. SGLT2 inhibitors (“gliflozins”) induce glycosuria and thereby reduce glycemia, body weight and blood pressure; they also reduce cardiovascular morbidity and mortality and slow the progression of kidney disease over the long term in patients with T2DM [150–153]. Their use in PLTDM is limited, as evidence of their safety in this context is scarce. Treatment with empagliflozin in rats with tacrolimus-induced diabetes reduced hyperglycemia and increased plasma insulin levels and islet size; it also enhanced glucose-induced insulin secretion in isolated pancreatic tissue [154]. In a case series of patients with diabetes after a heart transplant, empagliflozin reduced weight, furosemide dose and blood pressure, albeit changes in HbA1c were non-significant [155]. In a group of ten kidney recipients, canagliflozin improved glycemic control, weight and blood pressure [156]. Despite concurrent immunosuppression and glycosuria, there were no reports of urinary tract infections in either study.

#### Ambulatory Insulin Therapy

Due to the above-mentioned effects of CNI on insulin production and secretion by beta-cells, many patients with PLTDM require some sort of ambulatory insulin therapy to reach glycemic control [103, 157]. There are no randomized trials evaluating the use of insulin for the long-term management of PLTDM compared to other antidiabetic therapies, but a rational conduct is to continue insulin therapy in patients who are discharged with good glycemic control and no hypoglycemia. Nevertheless, it is crucial to make prompt adjustments in insulin dose and/or timing in the early post-discharge days, as many factors that increase insulin requirements during hospitalization will rapidly subside. For adjustments in basal insulin dose, the so called 3-0-3 scheme is a safe approach previously employed in several clinical trials [158, 159]. If fasting capillary blood glucose (CBG) is between 80 and 110 mg/dL, basal insulin is kept at the current dose. If fasting CBG is above 110 mg/dL, basal insulin dose is increased by 3 units, if fasting CBG is below 80 mg/dL, basal insulin is reduced by 3 units. In a patient with regular eating habits, adjustments in prandial insulin doses can be guided by 2-h postprandial CBG measurements: if values are above 140 mg/dL, the prandial insulin dose of the preceding meal can be increased by 1–2 units, while if values are below 70 mg/dL the dose should be reduced by 1–2 units [160].

### Impact of Immunosuppressive Regime on PTLDM Management

Despite the advent of safer and more effective immunosuppressive agents, steroids remain the most commonly used therapy for the induction and treatment of rejections, based on their efficacy and low cost. However, steroids have a well-recognized diabetogenic effect [161]. The tapering or withdrawal of steroids in order to diminish their metabolic effects is a controversial issue. In one clinical trial, 502 LT recipients with HBV infection were randomized to one of four steroid-minimization protocols (steroid withdrawal after 6 months, 3 months or 14 days, or complete avoidance of steroid therapy). After 3 years of follow-up, hyperglycemia and diabetes were significantly more frequent in patients on the two longer protocols, without any difference in patient survival, graft survival or chronic rejection among the four groups [162]. Another randomized trial of 82 adult LT patients found that “almost avoidance” of steroids (withdrawal within 24 h of receiving the transplant) was associated with much lower insulin requirements 1 week post-transplant compared to a more conventional regime (20.5 vs. 39.6 Units; *p* < 0.05) [163]. Other studies suggest that patients with older age or HCV as a cause for LT would specially benefit from an early steroid withdrawal protocol [164, 165]. Further, a retrospective analysis of 330 patients found no difference in PLTDM, hyperlipidemia or cardiovascular events at 4 years after the LT between patients with steroid withdrawal by 3 months or those with steroid withdrawal by 3–12 months post-procedure [166].

Of note, a recent meta-analysis of 16 clinical trials reported that steroid avoidance or withdrawal was accompanied by increased acute rejection (relative risk [RR] 1.33, 95% CI 1.08–1.64) and steroid-resistant rejection (RR 2.14, 95% CI 1.13–4.02), while achieving only a modest reduction in the risk of PLTDM (RR 0.81, 95% CI 0.66–0.99). These results clearly illustrate that glycemic control should not jeopardize patient and graft survival [167]. That does not mean, however, that steroids should be used liberally: one study assessed the impact of reducing the methylprednisolone dose from 10–15 mg per day to 5 mg per day during the first year in renal transplant recipients and found positive effects on insulin sensitivity [168]. Unfortunately, the optimal steroid dose needed to achieve a balance between prevention of LT rejection episodes and avoidance of glycemic impairment is still unknown.

Concerning CNIs, tapering of tacrolimus may have a positive effect on glucose tolerance [169, 170], but there are no trials directly aimed at assessing the influence of different CNI dosing regimens on glycemic control in PLTDM. A few trials have assessed metabolic outcomes after the change in treatment regimen from tacrolimus to cyclosporine, reporting notable benefits [171, 172]. Whether persistence of PLTDM is a sufficient indication for a change of immunosuppressant is still not well established.

## Conclusion

Post-liver transplant diabetes mellitus is a frequent condition with potentially disastrous consequences. PLTDM should be screened for, timely diagnosed and intensively managed. Clinicians in charge of caring for LT recipients should bear in mind key concepts about PLTDM (Table 2).

**Table 2:** Key concepts about post-liver transplant diabetes mellitus

Key concepts about PLTDM	
**What is PLTDM?** -PLTDM is any diabetes detected for the first time after a LT, once doses of immunosuppressants are tapered and stable	**How should PLTDM be managed?** - Optimal glycemic control during the early post-operative period is best achieved with carefully supervised, in-hospital intensive insulin regimes. Early control has a great impact on the long-term prognosis of transplant and patient -Patients with PLTDM, like any patient with diabetes, should be counseled on therapeutic lifestyle changes -For long-term PLTDM outpatient management, there is scarce evidence to support the choice of one antidiabetic agent over another -Metformin has a desirable mechanism of action in the context of PLTDM. Risk of lactic acidosis does not seem to be increased in LT patients -The potential risk for drug–drug interactions and risk of hypoglycemia make sulfonylureas not the optimal choice for PLTDM -Small studies have reported benefits with the use of thiazolidinediones in PLTDM, but the availability of these medications is limited in many parts of the world -DPP-4 inhibitors have limited efficacy, but a good safety record in PLTDM -Newer families of antidiabetics (GLP-1 analogues and SGLT2 inhibitors) have been assessed for PLTDM only in small case series so far. -Ambulatory insulin therapy is a feasible and effective way of controlling PLTDM, but dose adjustments must be done carefully and following clear, pre-established glycemic goals. -When adjusting immunosuppressive regimes, prevention of rejection should take preeminence over potentially adverse glycemic effects, which may be managed with a variety of strategies
**Why is PLTDM important?** -The number of LTs is rapidly increasing, and so is the frequency of PLTDM. -PLTDM impairs the survival of both graft and patient, and increases the risk of multiple undesirable outcomes in the short and long term	
**What causes PLTDM?** -Many patients develop PLTDM as a side effect of immunosuppressive therapy. Some others develop PLTDM because they bear the usual risk factors for type 2 diabetes. -The main mechanism by which CNIs cause PLTDM is by harming insulin-producing beta-cells. Glucocorticoids precipitate PLTDM mostly by inducing insulin resistance in target tissues	

*DDP-4* Dipeptidyl peptidase-4,* GLP-1* glucagon-like peptide 1,* PLTDM* Post-liver transplant diabetes mellitus,* SGLT2* sodium-glucose cotransporter type 2
